# Parametric study of lotus-type pore shape in solid subject to Henry's laws at interfaces

**DOI:** 10.1016/j.heliyon.2023.e18163

**Published:** 2023-07-22

**Authors:** B.Y. Lee, P.S. Wei

**Affiliations:** Department of Mechanical and Electro-Mechanical Engineering, National Sun Yat-Sen University, Kaohsiung 80424, Taiwan, ROC

**Keywords:** Porosity, Lotus-type pores, Pore shape, Solidification, Isolated pore, Sea ice

## Abstract

Mechanisms of the length and maximum radius of lotus-type or single pores in ice or nonmetals satisfied by Henry's law at gas-liquid interfaces dissolved by a gas during unidirectional solidification are rigorously investigated and supported by a Table from algebraic predictions involving different dimensionless working parameters. Lotus-type porous materials characterized by directional properties have been often used as functional materials in food, biomedical, and micro- and nano-technologies. Following previous work taking into account solute amount and transport within the pore, and concentration boundary layers on the advancing solid-liquid interface and bubble cap, and the Young-Laplace equation and Henry's law at liquid-gas interfaces, the algebraic study further provides a Table for a quantitative and extensive understanding of different mechanisms of length and maximum radius. Dimensionless parameters include solute transport parameters of Henry's law constant, mass transfer coefficient, partition coefficient, solute gas amount in imposed ambient, and solute transport parameter, and fluid and thermal parameters of solidification rate, imposed gas pressure, hydrostatic pressure, and geometrical parameter of inter-pore spacing. The controlling of the shapes of lotus-type pores is achieved by a good comparison between predicted maximum diameter and inter-pore spacing during freezing of water dissolved by oxygen gas.

## 1 Introduction

Materials containing gas porosity such as ice or nonmetals are encountered in various fields. For example, ice containing gases such as nitrogen and oxygen has been often used in food and fishery and preservation industries [1]. In view of strong oxidation, ice containing ozone has widely been used for deodorization, sterilization, purification of water, and prolongation of shelf–life foods [2,3]. Ozone gas can be dissolved into water with an ozone injection system, and fed into an ice making machine that will freeze water to produce ozonated ice. More than eighty years ago, research in the commercial fish industry was conducted in France that showed a 33% extension in shelf-life of fresh fish stored on ozonated ice when compared with ice produced from regular water in the holds of fishing vessels. Since gas amount in the ice is limited by gas solubility in water, Inada et al. [1] thus proposed a microbubble method to enhance ice containing gas pores. Microbubbles of a desired gas are dispersed, and then obtained images of the structural features of pores formed in the ice. The air amount in ice prepared by the microbubble method was three times higher than the solubility of air in water prepared by the conventional method. Matsumoto et al. [4] also studied pore formation during freezing water containing ozone micro-bubbles finding the effects of surfactant, inclination angles of the cooling plate on characteristics of ice containing oxygen micro-bubbles.

Ice containing pores filled with methane, oxygen or carbon dioxide has been often seen and well treated in geophysical sciences [[5], [6], [7]], and fundamental sciences [8,9]. In view of reflecting sunlight and obstructing thermal energy, growth and decay of ice in ponds, lakes and sea ice, which contain air pores are of interest. The occurrence of bubbles in fresh water ice relates to the gas amount in the pond/lake water prior to freezing over the surface. As freezing progresses, the interface concentration of dissolved gases surpasses a critical value, the water at the interface becomes supersaturated, and gas bubbles nucleate and grow to a visible size. When the sea water starts to freeze, most of salt amount is discharged from ice crystals into liquid below because the solubility limit of salt in the crystal lattice of ice is very small. However, a number of small volumes of saline water, called brine regions, and air pores remain in crystal grains of ice. Sea ice is thus also a porous material which is composed of pure ice crystals, brines and air inclusions. Crabeck et al. [6] discussed that formation of sea ice in winter can trap salts and $CO_{2}$ in brine from the ocean. $CO_{2}$ trapped with salt is transformed into solid rock. $CO_{2}$ also trapped in bubbles can rise from the bottom of the sea ice to the surface through the brine or air channels. Through the rising bubbles and sinking brine, the sea ice loses a lot of $CO_{2}$. As a result, when the sun comes back in the spring, the sea ice no longer holds as much $CO_{2}$.Sea ice can again absorb lots of $CO_{2}$ from the ambient. Sea ice thus can help the ocean to absorb $CO_{2}$ to fight climate change.

Shape development of the lotus-type pores in ice during upward freezing of water-carbon dioxide solutions were observed by Murakami and Nakajima [8]. Columnar pores were formed at low growth rates. The length of the columnar pores became shorter as the growth rate increased or the carbon dioxide concentration increased. The length of the columnar pores and periodic formation of pores were explained in terms of the rate of supply of carbon dioxide to the growing pores from the surrounding liquid. Yoshimura et al. [9] observed that lotus-type pores during solidification of water containing oxygen gas changed from egg, cylindrical into bifurcated cylindrical shapes as solidification rate increased. Theoretical analysis was conducted to scale solute transport in radial direction within the concentration boundary layer ahead of the advancing solid-liquid interface into pore, satisfied by Henry's law applicable to liquid containing a molecular solute gas with small concentration at the bubble cap. Diameter and inter-pore spacing were found to be inversely proportional product of solidification rate with square root of ambient pressure, agreeing with measurements. Other than proposing a universal phase diagram and simultaneous first-order ordinary differential equations [10], Wei and Huang [11] proposed a polynomial with three degrees to predict isolated pore shapes in solid by accounting for either solute transport from the pore across the bubble cap into surrounding liquid or that from the surrounding liquid into the pore in the early stage, denoted by Cases 1 and 2, respectively. Recently, Wei and Lee [12] combined Cases 1 and 2 interrelated by interaction angle and solute transport parameter to algebraically predict morphology of lotus-type pores. The predicted pore shapes were in good agreements with available experimental results during freezing of water filled with oxygen gas and carbon dioxide, and liquid copper imposed by hydrogen gas.

In this study, morphology of lotus-type pores satisfied by Young-Laplace equation and Henry's law at the bubble cap and top free surface during unidirectional solidification are parametrically presented. Instead of solving simultaneous first-order differential equations [10,11], this study extends previous work [12] to provide a table to get deep insight into understanding of dimensionless working parameters responsible for different shapes of lotus-type pores.

## 2 System model and analysis

**Fig. 1:**
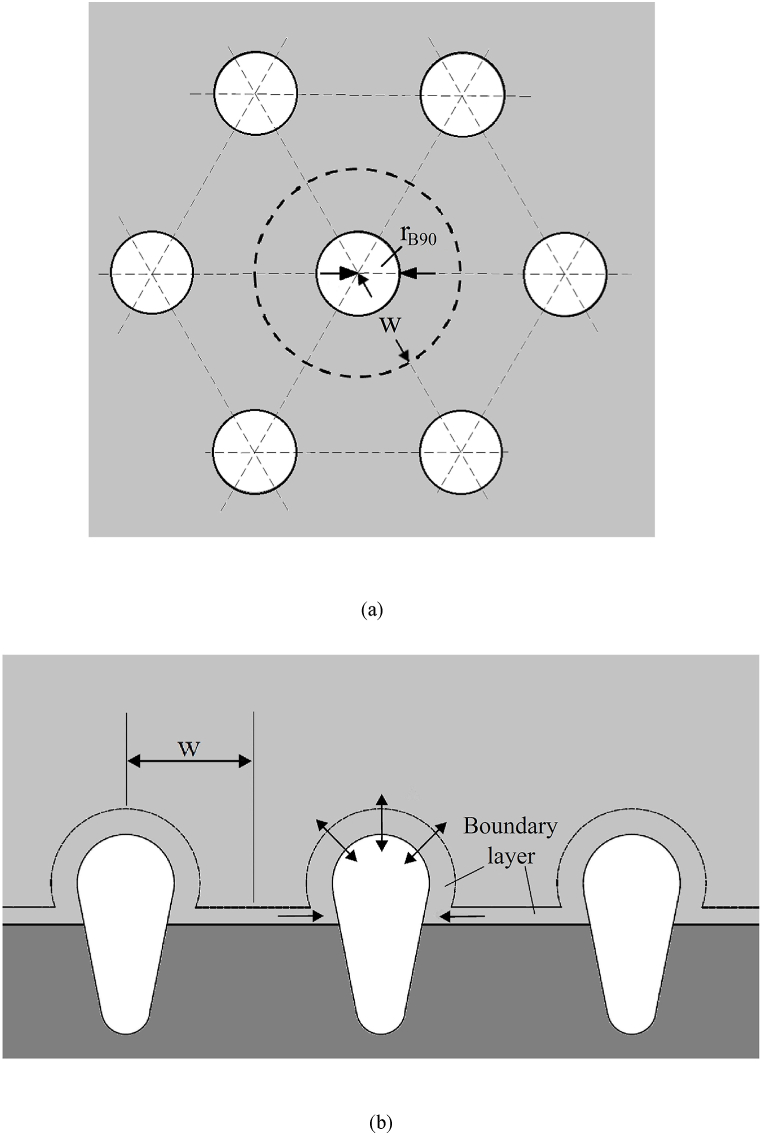
Entrapping lotus-type pores with (a) transverse and (b) longitudinal cross-sections [12].

Lotus-type pores with transverse and longitudinal cross-sections in maximum radius, ${\tilde{r}}_{B90}$, and inter-pore spacing, $\tilde{w}$, are illustrated in Fig. 1(a) and (b), respectively. Since solute rejected by the advancing solid-liquid interface results in supersaturation for bubble nucleation, concentration boundary layers occur along the advancing solid-liquid interface and on the bubble cap. The major assumptions made are as follows:

1. The pore grows unidirectionally. Furthermore, the variations of transport variables and pore shape in the circumferential direction are much smaller than those in the axial direction. This can be confirmed by the lotus-type pores in cylindrical shape (see Fig. 1(a)).
2. The tiny bubble with spherical caps at the top and bottom is characterized by a small Bond number. Bond number $Bo\equiv {\tilde{\rho }}_{l}g{\tilde{R}}_{0}^{2}/\sigma$ representing the ratio between hydrostatic pressure and capillary pressure can be as small as $10^{-5}$ in most cases, based on a typical bubble radius ${\tilde{R}}_{0}$ = $10^{-5}$ m.
3. The tiny pore is in a lumped system.
4. Fluid flow is dominated by force convection rather than free convection driven by heat transfer or mass transfer [13]. Velocity of free convection driven by heat transfer in the thermal boundary layer can be scaled by considering balance between viscous force and free convection due to heat transfer, $\mu v_{T,NC}/\delta_{T}^{2}∼\rho \beta_{T}g\Delta T$, and thermal convection and conduction, $v_{T,NC}/H∼\alpha_{l}/\delta_{T}^{2}$. Velocity of free convection driven by heat transfer thus yields [14]

(1) $$v_{T,NC}∼\frac{\alpha_{l}}{H}Ra_{T}^{1/2}∼5\times 10^{-5}\;m/s$$

where Rayleigh number due to temperature $Ra_{T}\equiv \rho \beta_{T}g\Delta TH^{3}/\alpha_{l}\mu ∼10^{-5}$ based on temperature expansion coefficient $\beta_{T}$ ∼$5\times 10^{-5}$ 1/K, dynamic viscosity $\mu ∼1.7\times 10^{-3}\;kg/m-s$, and thermal diffusivity $\alpha_{l}∼1.3\times 10^{-7}m^{2}/s$ of water [14], length of the physical domain $H∼10^{-5}$ m and temperature difference ΔT ∼1 K. Similarly, considering balance between viscous stress and free convection due to concentration $\mu v_{C,NC}/\delta_{C}^{2}∼\rho \beta_{C}g\Delta C$ and balance between convection and diffusion $v_{C,NC}/H∼D_{l}/\delta_{C}^{2}$ in the concentration boundary layer gives velocity of free convection driven by mass transfer [14]

(2) $$v_{C,NC}∼\frac{D_{l}}{H}Ra_{C}^{1/2}∼3\times 10^{-6}\;m/s$$

where Rayleigh number due to concentration $Ra_{C}\equiv \rho \beta_{C}g\Delta CH^{3}/\mu D_{l}∼10^{-5}$ , based on concentration expansion coefficient of water due to oxygen gas or carbon dioxide $\beta_{C}∼10^{-4}m^{3}/\text{kg}$, solute diffusivity $D_{l}∼10^{-9}m^{2}/s$ [14], and difference in solute concentration ΔC ∼10 mol/$m^{3}$. Referring to Eqs. (1), (2) indicates that velocity of free convection driven by either heat transfer or mass transfer is much smaller than velocity of force convection greater than 0.01 m/s [13].

5. Marangoni force due to temperature (namely, thermocapillary force), or concentration can be conditionally neglected. Consider viscous stress to be balanced by thermocapillary force, $\mu v_{T,FC}/\delta_{FC}∼\left(d\sigma /dT\right)\Delta T/H$, and inertia force to be balanced by viscous stress, $\rho v_{T,FC}^{2}/H∼\mu v_{T,FC}/\delta_{FC}^{2}$ [15]. It then gives velocity due to thermocapillary force

(3) $$v_{T,FC}∼{Ma}_{T}^{2/3}\;{\mathrm{Pr}}^{1/3}\alpha_{l}/H∼0.1\;m/s$$

where Marangoni number $Ma_{T}\equiv -\left(d\sigma /dT\right)\Delta TH/\mu \alpha_{l}$ ∼7, Prandtl number $\mathrm{Pr}\equiv \mu /\rho \alpha_{l}$ ∼13 [14], and surface tension coefficient due to temperature dσ/dT ∼ $1.5\times 10^{-4}$ N/m-K [16]. Similarly, viscous stress balanced by Marangoni force due to concentration, $\mu v_{C,FC}/\delta_{FC}∼\left(d\sigma /dC\right)\Delta C/H$, and inertia force balanced by viscous stress $\rho v_{C,FC}^{2}/H∼\mu v_{C,FC}/\delta_{FC}^{2}$ gives velocity induced by Marangoni force due to concentration

(4) $$v_{C,FC}∼Ma_{C}^{2/3}Sc^{1/3}D_{l}/H∼0.01\;m/s$$

where Marangoni number $Ma_{C}\equiv -\left(d\sigma /dC\right)\Delta CH/\mu D$ ∼60, surface tension coefficient due to concentration $d\sigma /dC∼10^{-6}$ $Nm^{2}/mol$ [17,18], and Schmidt number Sc≡μ/ρD ∼450 [14]. Eqs. (3), (4) indicates that Marangoni force can be conditionally considered, provided that velocity due to force convection is not much higher than 0.1 m/s.

6. The weakness of this model can be attributed to the strong effects of thermocapillary convection due to temperature on fluid flow, as stated in item 5. As a result, liquid pressure in the Young-Laplace equation deviates from hydrostatic pressure, which is only a function of depth in the liquid layer and gravitational acceleration. However, integration method used in this model to evaluate solute content in the system is acceptable, even though convection is accounted [19].
7. Tangential and normal stresses exerted by fluid flow are thus negligible in this static system. Liquid pressure is identical to hydrostatic pressure.
8. Inter-pore spacing which is a result of constitutional supercooling is specified [20].

**Fig. 2:**
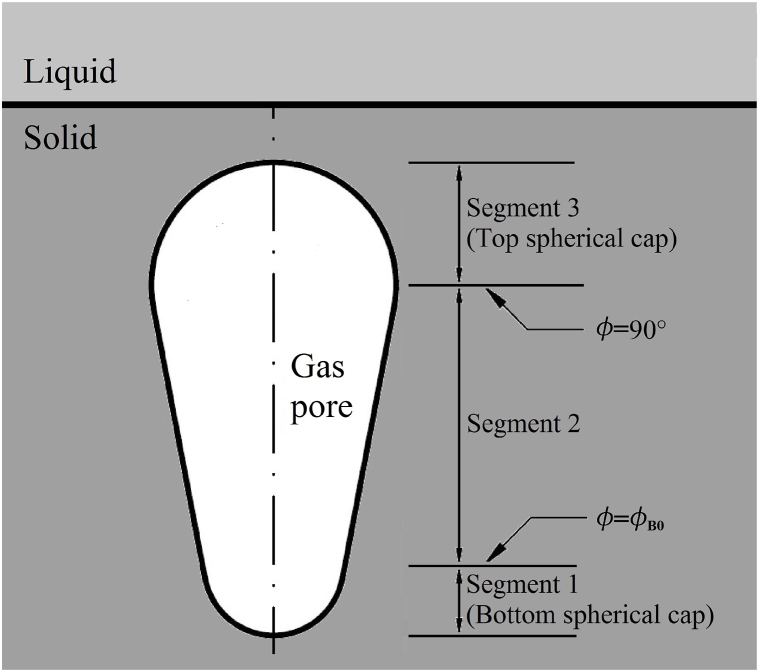
Three segments to predict pore shape in solid [12].

With the above assumption, the entrapped bubble in solid is divided into three segments, defined by initial contact angle, and inclination angle or contact angle of 90°, as sketched by Fig. 2 [12]. The pore shape is closely related and described by the second segment.

### 2.1 Shape of the second segment

Description of the second segment can be a polynomial with five degrees

$$r=a+b\left(\frac{s}{l_{90}}\right)+c{\left(\frac{s}{l_{90}}\right)}^{2}+d{\left(\frac{s}{l_{90}}\right)}^{3}+e{\left(\frac{s}{l_{90}}\right)}^{4}+f{\left(\frac{s}{l_{90}}\right)}^{5}$$

(5) $$for\;90^{0}\leq \phi \leq \phi_{B0}$$

where coefficients

(6) $$a=\sin\phi_{B0}$$

(7) $$b=-\frac{l_{90}}{\tan\;\phi_{B0}}$$

(8) $$c=10\left(r_{B90}-\sin\phi_{B0}\right)+\frac{4l_{90}}{tan\phi_{B0}}$$

(9) $$d=-2c+\frac{2l_{90}}{\tan\;\phi_{B0}}=-20\left(r_{B90}-\sin\;\phi_{B0}\right)-\frac{6l_{90}}{\tan\;\phi_{B0}}$$

(10) $$e=-\frac{c}{2}-d=15\left(r_{B90}-\sin\;\phi_{B0}\right)+\frac{4l_{90}}{\tan\;\phi_{B0}}$$

(11) $$f=-\frac{d}{10}-\frac{2e}{5}=-4\left(r_{B90}-\sin\;\phi_{B0}\right)-\frac{l_{90}}{\tan\phi_{B0}}$$

Eqs. (6), (7), (8), (9), (10), (11) are derived by satisfying *r =* $\sin\;\phi_{B0}$*, dr/ds = -1/* $\tan\;\phi_{B0}$ *at s = 0,* and *r =* $r_{B90}$*, dr/ds =* $d^{2}r/ds^{2}$ = $d^{3}r/ds^{3}$ = *0* at *s =* $l_{90}$ *.* Other degree of polynomials can be found in previous work [21].

### 2.2 Solute conservation

Determination of length and maximum radius of the second segment in Eqs. (5), (6), (7), (8), (9), (10), (11) accounts for solute conservation in a controlled system.

#### 2.2.1 Total solute conservation

**Fig. 3:**
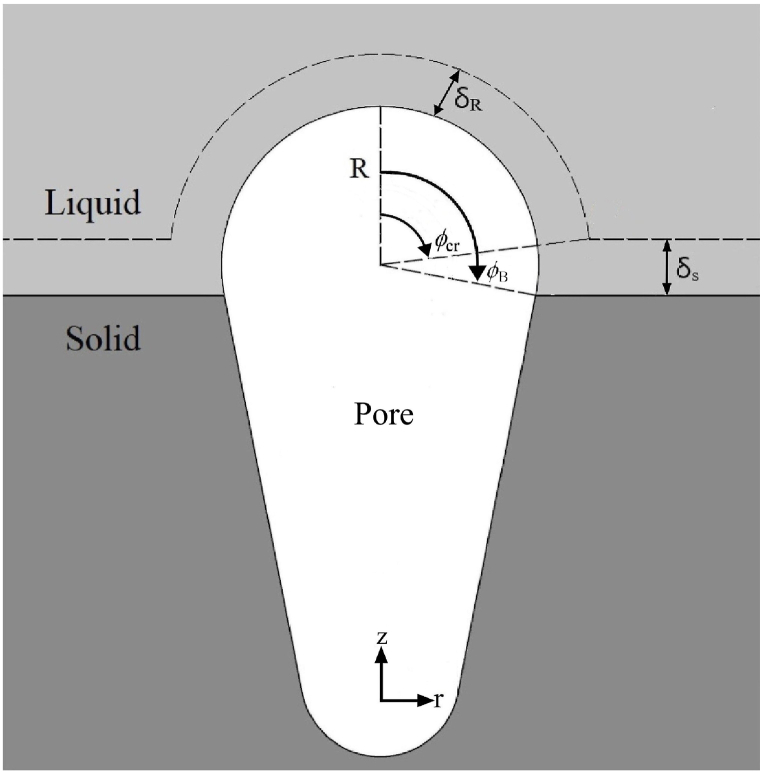
Geometrical and solute transport model [12].

Solute molecules in the controlled system subject to adiabatic boundary conditions are conserved and given by

(12) $${\tilde{\rho }}_{g}\tilde{V}+\int_{\tilde{R}}^{\tilde{R}+{\tilde{\delta }}_{R}}\left({\tilde{C}}_{l}-{\tilde{C}}_{\infty }\right)2\pi {\tilde{r}}^{2}\left(1-\cos\phi_{cr}\right)d\tilde{r}+\int_{\tilde{s}}^{\tilde{s}+{\tilde{\delta }}_{s}}\left({\tilde{C}}_{l}-{\tilde{C}}_{\infty }\right)\pi \left({\tilde{w}}^{2}-{\tilde{R}}^{2}\;\sin^{2}\phi_{B}\right)d\tilde{z}=const\text{.}\;={\tilde{\rho }}_{g0}{\tilde{V}}_{0}+\int_{{\tilde{R}}_{0}}^{{\tilde{R}}_{0}+{\tilde{\delta }}_{R0}}\left({\tilde{C}}_{l}-{\tilde{C}}_{\infty }\right)2\pi {\tilde{r}}^{2}\left(1-\cos\;\phi_{cr0}\right)d\tilde{r}+\int_{0}^{{\tilde{\delta }}_{s0}}\left({\tilde{C}}_{l}-{\tilde{C}}_{\infty }\right)\pi \left({\tilde{w}}^{2}-{\tilde{R}}_{0}^{2}\;\sin^{2}\phi_{B0}\right)d\tilde{z}$$

where the left-hand side represents conserved total solute molecules including solute molecules within the entrapping bubble, and boundary layers on the cap and advancing solid-liquid interface, respectively. Intersection angle $\phi_{cr}$ between two boundary layers is illustrated in Fig. 3. Integrals in Eq. (12) can be evaluated by substituting concentration profiles within boundary layers on the cap and advancing solid-liquid interface [12]

(13) $$\frac{{\tilde{C}}_{l}-{\tilde{C}}_{\infty }}{{\tilde{C}}_{c}-{\tilde{C}}_{\infty }}={\left(1-\frac{\tilde{r}-\tilde{R}}{{\tilde{\delta }}_{R}}\right)}^{2},\frac{{\tilde{C}}_{l}-{\tilde{C}}_{\infty }}{{\tilde{C}}_{ls}-{\tilde{C}}_{\infty }}={\left(1-\frac{\tilde{z}-\tilde{s}}{{\tilde{\delta }}_{s}}\right)}^{2}$$

where concentration boundary layer thicknesses [11,12].

(14) $${\tilde{\delta }}_{R}=\frac{2{\tilde{D}}_{l}}{{\tilde{h}}_{D}},{\tilde{\delta }}_{s}=\frac{2{\tilde{D}}_{l}}{\tilde{U}}$$

Pore volume in Eq. (12) is

(15) $$\tilde{V}=\frac{\pi }{3}{\tilde{R}}_{0}^{3}\left(2-\cos\;\phi_{B0}\right){\left(1+\cos\;\phi_{B0}\right)}^{2}+\int_{0}^{\tilde{s}}\pi {\tilde{r}}_{B}^{2}ds^{'}+\frac{\pi }{3}{\tilde{R}}^{3}\left(2+\cos\;\phi_{B}\right){\left(1-\cos\;\phi_{B}\right)}^{2}$$

where integral can be approximated by

(16) $$\int_{0}^{\tilde{s}}\pi {\tilde{r}}_{B}^{2}ds^{'}\approx \pi \left(\frac{2}{3}{\tilde{r}}_{B}^{2}+\frac{1}{3}{\tilde{R}}_{0}^{2}\;\sin^{2}\phi_{B0}\right)\tilde{s}$$

Henry's law at the bubble cap and top surface are, respectively [22].

(17) $${\tilde{C}}_{c}=\frac{{\tilde{p}}_{g}}{{\tilde{K}}_{Hc}},{\tilde{C}}_{\infty }=\frac{\gamma {\tilde{p}}_{a}}{{\tilde{K}}_{H\infty }}$$

Dimensionless length of the second segment by substituting Eqs. (13), (14), (15), (16), (17) into Eq. (12) gives

(18) $$l_{90\leq \phi \leq \phi_{B0}}=\frac{1}{\frac{2}{3}r_{B90}^{2}+\frac{1}{3}\sin^{2}\phi_{B0}}\{\frac{1}{\frac{2}{r_{B90}}+p_{g0}-2}\{\frac{4p_{g0}}{3}+\left(\frac{p_{g0}}{K_{Hc}}-BoC_{\infty }\right)F_{1}\left(\phi_{B0},h_{D0},U_{0}\right)\;-\left[\frac{1}{K_{Hc}}\left(\frac{2}{r_{B90}}+p_{g0}-2\right)-BoC_{\infty }\right]F_{2}\left(r_{B90},h_{D90},U_{90}\right)\;+BoC_{\infty }\left[F_{4}\left(\phi_{B0},k_{p0},r_{B0},w,U_{0}\right)-F_{5}\left(k_{p90},r_{B90},w,U_{90}\right)\right]\}-F_{3}\left(\phi_{B0},r_{B90}\right)\}$$

where dimensionless functions

(19) $$F_{1}\left(\phi_{B0},h_{D0},U_{0}\right)\equiv \frac{2}{30}\left(1-\frac{\frac{2}{U_{0}}+\cos\;\phi_{B0}}{1+\frac{2}{h_{D0}}}\right)\left[{\left(\frac{2}{h_{D0}}\right)}^{3}+5{\left(\frac{2}{h_{D0}}\right)}^{2}+10\frac{2}{h_{D0}}\right]$$

(20) $$F_{2}\left(r_{B90},h_{D90},U_{90}\right)\equiv \frac{2r_{B90}^{3}}{30}\left(1-\frac{\frac{2}{U_{90}r_{B90}}}{1+\frac{2}{h_{D90}r_{B90}}}\right)\left[{\left(\frac{2}{h_{D90}r_{B90}}\right)}^{3}+5{\left(\frac{2}{h_{D90}r_{B90}}\right)}^{2}+10\frac{2}{h_{D90}r_{B90}}\right]$$

(21) $$F_{3}\left(\phi_{B0},r_{B90}\right)\equiv \frac{2r_{B90}^{3}}{3}+\frac{1}{3}\left(2-\cos\;\phi_{B0}\right){\left(1+\cos\;\phi_{B0}\right)}^{2}$$

(22) $$F_{4}\left(\phi_{B0},k_{p0},r_{B0},w,U_{0}\right)\equiv \left(\frac{1}{k_{p0}}-1\right)\left(w^{2}-\sin^{2}\phi_{B0}\right)\frac{2}{3U_{0}}$$

(23) $$F_{5}\left(k_{p90},r_{B90},w,U_{90}\right)\equiv \left(\frac{1}{k_{p90}}-1\right)\left(w^{2}-r_{B90}^{2}\right)\frac{2}{3U_{90}}$$

and

(24) $$p_{g0}=Bo\left(h_{B0}-z_{B0}\right)+p_{a}+2$$

(25) $$BoC_{\infty }=\frac{\gamma p_{a}}{K_{H\infty }}$$

Eqs. (24), (25) are, respectively, the Young-Laplace equation in the pore at initial time and Henry's law at the top surface.

#### 2.2.2 Solute concentration in pore

Changing rate of solute amount in the entrapping bubble at contact angle of 90° yields

(26) $$\frac{{\tilde{p}}_{g}}{{\tilde{R}}_{u}{\tilde{T}}_{m}}\pi {\tilde{r}}_{B90}^{2}{\tilde{U}}_{90}=2\pi {\tilde{r}}_{B90}^{2}\left(1-\frac{\frac{2{\tilde{D}}_{l}}{{\tilde{U}}_{90}}}{{\tilde{r}}_{B90}+\frac{2{\tilde{D}}_{l}}{{\tilde{h}}_{D90}}}\right){\tilde{h}}_{D90}\left({\tilde{C}}_{\infty }-\frac{{\tilde{p}}_{g}}{{\tilde{K}}_{Hc}}\right)+\pi \eta \left({\tilde{w}}^{2}-{\tilde{r}}_{B90}^{2}\right){\tilde{U}}_{90}{\tilde{C}}_{\infty }\left(\frac{1}{k_{p90}}-1\right)$$

where terms on the right-hand stand for solute transport from boundary layers on the cap and advancing solid-liquid interface, respectively. The dimensionless maximum radius from Eq. (26) thus yields

(27) $$A_{H}r_{B90}^{3}+B_{H}r_{B90}^{2}+C_{H}r_{B90}+D_{H}=0$$

with

(28) $$A_{H}\equiv 2-p_{g0}+a_{H}-\eta BoC_{\infty }\left(\frac{1}{k_{p90}}-1\right)$$

(29) $$B_{H}\equiv \frac{2A_{H}}{h_{D90}}-2\left(1+\frac{2h_{D90}}{U_{90}K_{Hc}}\right)-\frac{2a_{H}}{U_{90}}$$

(30) $$C_{H}\equiv \frac{4}{U_{90}}\frac{2h_{D90}}{U_{90}K_{Hc}}+\eta w^{2}BoC_{\infty }\left(\frac{1}{k_{p90}}-1\right)-\frac{4}{h_{D90}}\left(1+\frac{2h_{D90}}{U_{90}K_{Hc}}\right)$$

(31) $$D_{H}\equiv \frac{2}{h_{D90}}\eta BoC_{\infty }\left(\frac{1}{k_{p90}}-1\right)w^{2}$$

(32) $$a_{H}\equiv \frac{2h_{D90}}{U_{90}K_{Hc}}\left(2-p_{g0}+K_{Hc}BoC_{\infty }\right)$$

Eq. (27) with algebraic coefficients from Eqs. (28), (29), (30), (31), (32) can be exactly and analytically obtained by fundamental mathematics.

## 3 Results and discussion

Lotus-type pores in solid are algebraically predicted for different independent dimensionless working parameters based on selected typical values of $K_{Hc}$ = $K_{H\infty }$ = *34,* $U_{0}$ = *0.11,* $U_{90}$ = *0.035,* $k_{p0}$ = *0.0088,* $k_{p90}$ = *0.008,* $h_{D0}$ = *0.09,* $h_{D90}$ = *0.03,* $p_{a}$ = *790,* $Bo\left(h_{B0}-z_{B0}\right)$ = *7.86,* γ = *1, w = 2.5,* $\phi_{B0}$ = *140°*, and η = *1*. Instead of solving simultaneous first-order ordinary equations [10], algebraic equations explicitly gain deep insight into mechanisms of length and maximum radius of lotus-type or single pores. Henry's law constants are allowed to be different at the bubble cap and top free surface, since the latter is higher than the former due to a decrease in surface curvature [23]. Solidification rates, mass transfer coefficients and partition coefficients can be varied during solidification. Since concentration boundary layer thickness increases as a result of decrease in solidification rate, mass transfer coefficient on the bubble cap decreases in the course of solidification, as can be seen from Eq. (14). Because effective partition coefficient increases with solidification rate [24], partition coefficient at inclination angle of 90° is specified to be less than that at initial contact angle. Partition coefficient can also be given by

(33) $$k_{p}=\frac{k_{eq}+\frac{{\tilde{\delta }}_{s}\tilde{U}}{{\tilde{D}}_{l}}}{1+\frac{{\tilde{\delta }}_{s}\tilde{U}}{{\tilde{D}}_{l}}}$$

Substituting Eq. (14) into Eq. (33) shows that partition coefficient is relatively constant and greater than equilibrium partition coefficient. Since partition coefficient is defined by the ratio between solute concentrations in solid and liquid at the advancing solid-liquid interface, its value can be affected by convection, boundary layers, interface morphology, etc. [24].

The length of lotus-type pores can be approximated by the length of the second segment determined by Eq. (18), governed by solute in the boundary layer on the entrapping bubble at initial time and contact angle of 90°, namely, $\left(p_{g0}/K_{Hc}-BoC_{\infty }\right)F_{1}\left(\phi_{B0},h_{D0},U_{0}\right)$, $\left(p_{g90}/K_{Hc}-BoC_{\infty }\right)F_{2}\left(r_{B90},h_{D90},U_{90}\right)$, and solute in boundary layer on the advancing solid-liquid interface at initial time and contact angle of 90°, $BoC_{\infty }F_{4}\left(\phi_{B0},k_{p0},r_{B0},w,U_{0}\right)$, and $BoC_{\infty }$$F_{5}\left(k_{p90},r_{B90},w,U_{90}\right)$, respectively. Functions $F_{1}\left(\phi_{B0},h_{D0},U_{0}\right)$, $F_{2}\left(r_{B90},h_{D90},U_{90}\right)$, $F_{4}\left(\phi_{B0},k_{p0},r_{B0},w,U_{0}\right)$, and $F_{5}\left(k_{p90},r_{B90},w,U_{90}\right)$ are, respectively, related to thicknesses of concentration boundary layers on the cap and advancing liquid-solid interface (see Eqs. (19), (20), (22), (23)). Therefore, pore length increases as solute amount in boundary layers on the cap and advancing solid-liquid interface at initial time increases, whereas that at inclination angle of 90° decrease. Solute in top and bottom spherical caps at contact angle of 90° is referred to $p_{g90}F_{3}\left(\phi_{B0},r_{B90}\right)$ from Eq. (21). The maximum radius is, however, calculated from Eq. (27) related to time change in solute amount in the entrapping bubble as a result of solute transfer rates through the cap from boundary layers at contact angle of 90°.

**Fig. 4:**
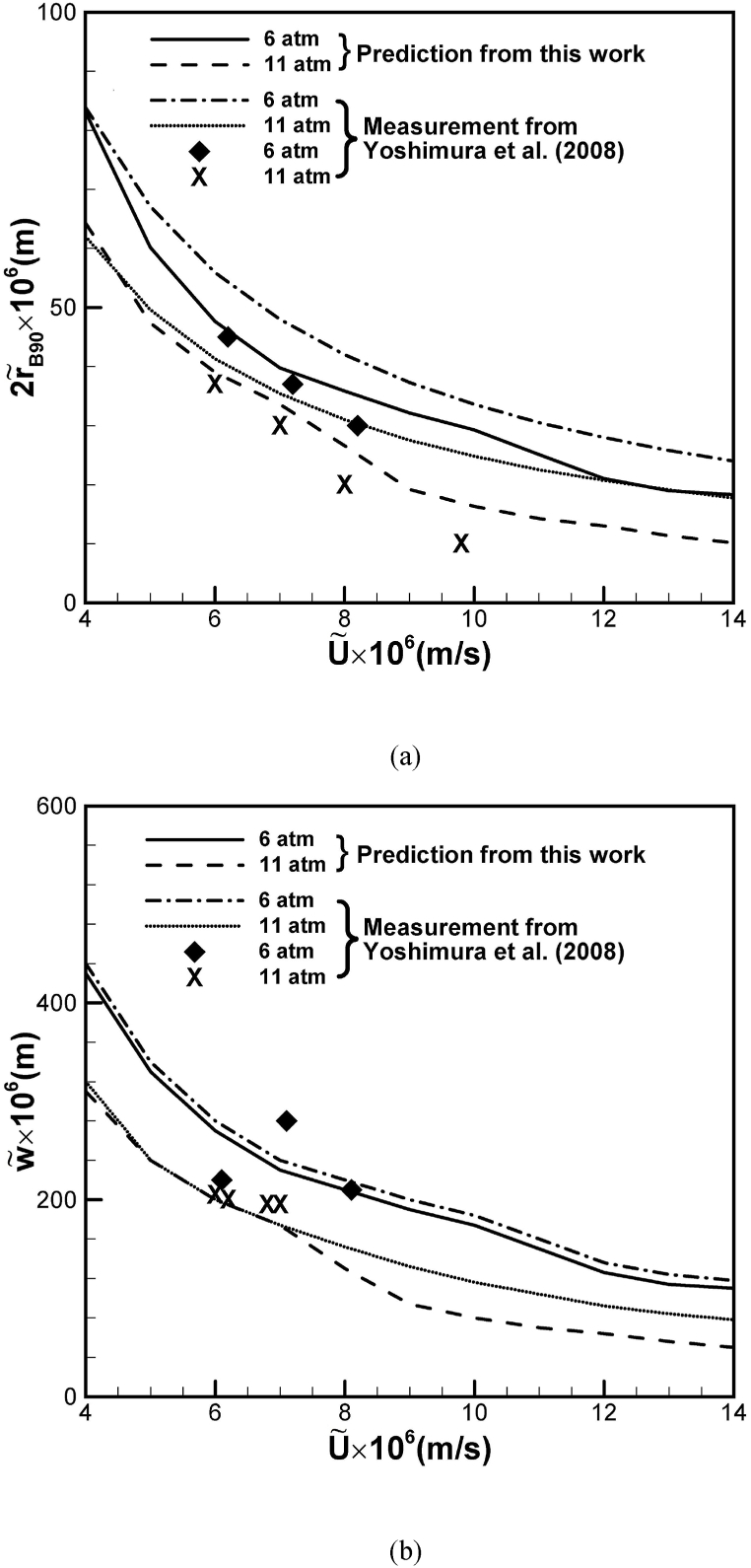
Prediction and measurement of (a) maximum diameter and (b) inter-pore spacing versus solidification rate during freezing of water dissolved by oxygen gas with different pressures [12].

**Fig. 5:**
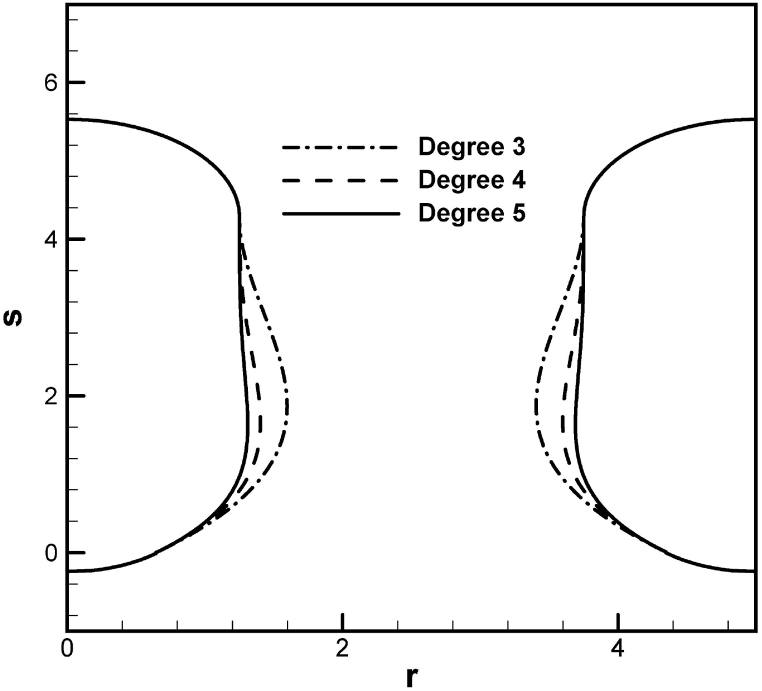
Predicted lotus-type pore shapes for different degrees of polynomials.

Predicted maximum diameter and inter-pore spacing versus solidification rate during unidimensional freezing of water subject to different imposed oxygen gas pressures at the top free surface agree with available experimental results [9], as presented in Fig. 4(a) and (b), respectively. Increases in solidification speed and imposed solute gas pressure at the top surface decrease the maximum diameter and inter-pore spacing. Polynomials with three, four and five degrees to predict morphology of lotus-type pores are shown in Fig. 5. It can be seen that a polynomial with five degrees used in this work is sufficiently accurate to delineate morphology of lotus-type pores.

**Fig. 6:**
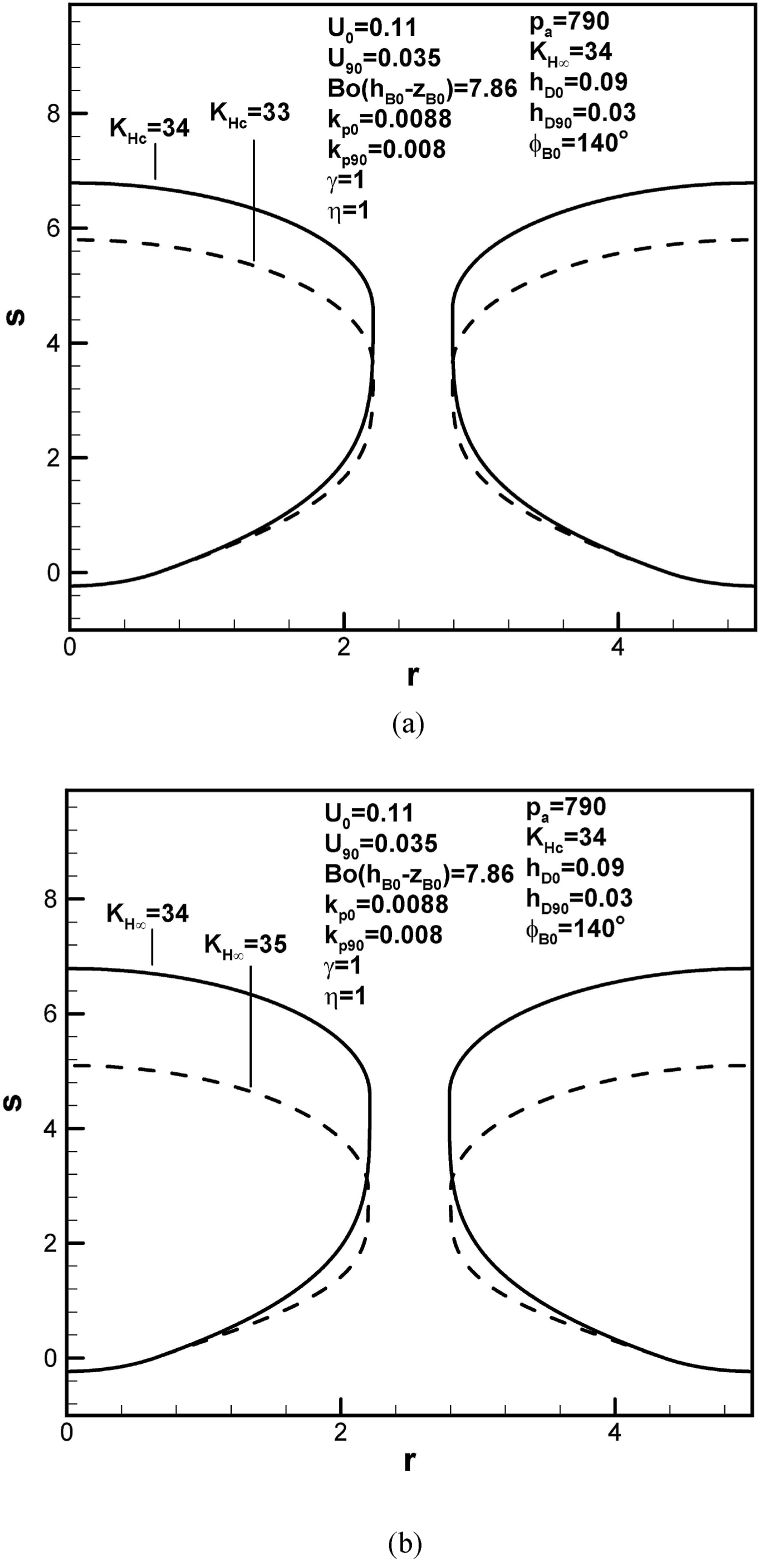
Predicted lotus-type pore shapes affected by Henry's law constants at (a) bubble cap, and (b) top free surface.

Dimensionless length of lotus-type pores increases with dimensionless Henry's law constant at the cap, as can be seen from Fig. 6(a). Referring to Table 1, this is attributed to decrease in solute amount in the boundary layer on the cap at contact angle of 90°. Difference in concentrations across the boundary layer between the cap and top free surface decreases as Henry's law constant at the cap increase. On the other hand, pore length increases as Henry's law constant at the top surface decreases, as shown in Fig. 6(b). This is because of increase in solute amount in the boundary layer on the advancing solid-liquid interface at initial time. Predicted result also agrees with that of a single isolated pore and specification of solute concentration in liquid far from the pore [[10], [11], [12]].

**Table 1:** Functions for determination of length of pores.

	$r_{B90}$	$l_{90}$	$\frac{4p_{g0}}{3}$	$\begin{array}{l} \left(\frac{p_{g0}}{K_{Hc}}-BoC_{\infty }\right)\\ F_{1}\left(\phi_{B0},h_{D0},U_{0}\right) \end{array}$	$\begin{array}{l} \left(\frac{p_{g90}}{K_{Hc}}-BoC_{\infty }\right)\\ F_{2}\left(r_{B90},h_{D90},U_{90}\right) \end{array}$	$\begin{array}{l} BoC_{\infty }\\ F_{4}\left(\phi_{B0},k_{p0},r_{B0},w,U_{0}\right) \end{array}$	$\begin{array}{l} BoC_{\infty }\\ F_{5}\left(k_{p90},r_{B90},w,U_{90}\right) \end{array}$	$p_{g90}$ $F_{3}\left(\phi_{B0},r_{B90}\right)$
Standard	*2.212*	*4.58*	$1.07\times 10^{3}$	*66*	$1.02\times 10^{3}$	$9.26\times 10^{4}$	$7.45\times 10^{4}$	$5.8\times 10^{3}$
*w = 3*	*2.655*	*4.94*	$1.07\times 10^{3}$	*66*	$1.07\times 10^{3}$	$1.36\times 10^{5}$	$1.07\times 10^{5}$	$10^{4}$
$U_{0}$ = *0.1*	*2.212*	*7.98*	$1.07\times 10^{3}$	*45.4*	$1.02\times 10^{3}$	$1.02\times 10^{5}$	$7.45\times 10^{4}$	$5.8\times 10^{3}$
$U_{90}$ = *0.04*	*2.212*	*7.78*	$1.07\times 10^{3}$	*66*	$1.64\times 10^{3}$	$9.26\times 10^{4}$	$6.52\times 10^{4}$	$5.8\times 10^{3}$
$h_{D0}$ = *0.05*	*2.212*	*4.85*	$1.07\times 10^{3}$	*805*	$1.02\times 10^{3}$	$9.26\times 10^{4}$	$7.45\times 10^{4}$	$5.8\times 10^{3}$
$h_{D90}$ = *0.025*	*2.212*	*3.82*	$1.07\times 10^{3}$	*66*	$3.07\times 10^{3}$	$9.26\times 10^{4}$	$7.45\times 10^{4}$	$5.8\times 10^{3}$
$K_{Hc}$ = *33*	*2.212*	*3.59*	$1.07\times 10^{3}$	*228*	$3.84\times 10^{3}$	$9.26\times 10^{4}$	$7.45\times 10^{4}$	$5.8\times 10^{3}$
$K_{H\infty }$ = *35*	*2.205*	*2.9*	$1.07\times 10^{3}$	*217*	$3.65\times 10^{3}$	$8.99\times 10^{4}$	$7.4\times 10^{4}$	$5.75\times 10^{3}$
$p_{a}$ = *500*	*2.211*	*4.21*	*680*	*66*	$1.02\times 10^{3}$	$5.86\times 10^{4}$	$4.74\times 10^{4}$	$3.69\times 10^{3}$
η=0.9	*2.186*	*2.38*	$1.07\times 10^{3}$	*66*	$1.02\times 10^{3}$	$9.26\times 10^{4}$	$8.08\times 10^{4}$	$5.6\times 10^{3}$
γ = *0.97*	*2.205*	*2.82*	$1.07\times 10^{3}$	*225*	$3.78\times 10^{3}$	$8.98\times 10^{4}$	$7.4\times 10^{4}$	$5.75\times 10^{3}$
γ = *1.03*	*2.219*	*6.33*	$1.07\times 10^{3}$	*−92.7*	$-1.74\times 10^{3}$	$9.54\times 10^{4}$	$7.49\times 10^{4}$	$5.86\times 10^{3}$
$\phi_{B0}=120^{o}$	*2.212*	*2.48*	$1.07\times 10^{3}$	*63*	$1.02\times 10^{3}$	$8.72\times 10^{4}$	$7.45\times 10^{4}$	$5.93\times 10^{3}$
$Bo\left(h_{B0}-z_{B0}\right)=11.79$	*2.211*	*4.29*	$1.07\times 10^{3}$	*92.4*	$1.48\times 10^{3}$	$9.26\times 10^{4}$	$7.47\times 10^{4}$	$5.82\times 10^{3}$
${k_{p}}_{0}$ = *0.008*	*2.212*	*8.02*	$1.07\times 10^{3}$	*66*	$1.02\times 10^{3}$	$1.02\times 10^{5}$	$7.45\times 10^{4}$	$5.8\times 10^{3}$
${k_{p}}_{0}$ = *0.0078*	*2.212*	*8.99*	$1.07\times 10^{3}$	*66*	$1.02\times 10^{3}$	$1.05\times 10^{5}$	$7.45\times 10^{4}$	$5.8\times 10^{3}$
${k_{p}}_{90}$ = *0.0088*	*2.188*	*5.34*	$1.07\times 10^{3}$	*66*	$1.02\times 10^{3}$	$9.26\times 10^{4}$	$7.29\times 10^{4}$	$5.62\times 10^{3}$
${k_{p}}_{90}$ = *0.009*	*2.182*	*5.53*	$1.07\times 10^{3}$	*66*	$1.02\times 10^{3}$	$9.26\times 10^{4}$	$7.25\times 10^{4}$	$5.58\times 10^{3}$

*Standard: w = 2.5, $U_{0}$ = 0.11, $U_{90}$ = 0.035, $h_{D0}$ = 0.09, $h_{D90}$ = 0.03, $K_{Hc}$ = $K_{H\infty }$ = 34, $p_{a}$ = 790, η = γ = 1, $Bo\left(h_{B0}-z_{B0}\right)$ = 7.86, $\phi_{B0}$ = $140^{o}$, ${k_{p}}_{0}$ = 0.0088, ${k_{p}}_{90}$ = 0.008.

**Fig. 7:**
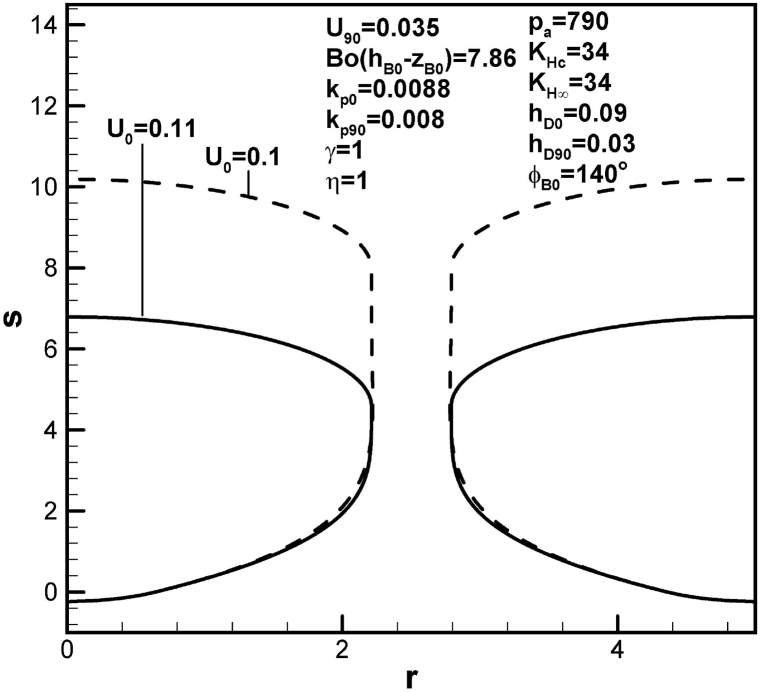
Predicted lotus-type pore shapes affected by dimensionless solidification rates at time corresponding to initial time.

The effects of dimensionless solidification rate at the initial time on the size of lotus-type pores are shown in Fig. 7. An increase in dimensionless solidification rate at the initial time from 0.1 to 0.11 for solidification rate at contact angle of 90° of 0.035 is seen to decrease pore length. This is because of decrease in solute amount in the boundary layer on the advanced solid-liquid interface at initial time (see Table 1).

**Fig. 8:**
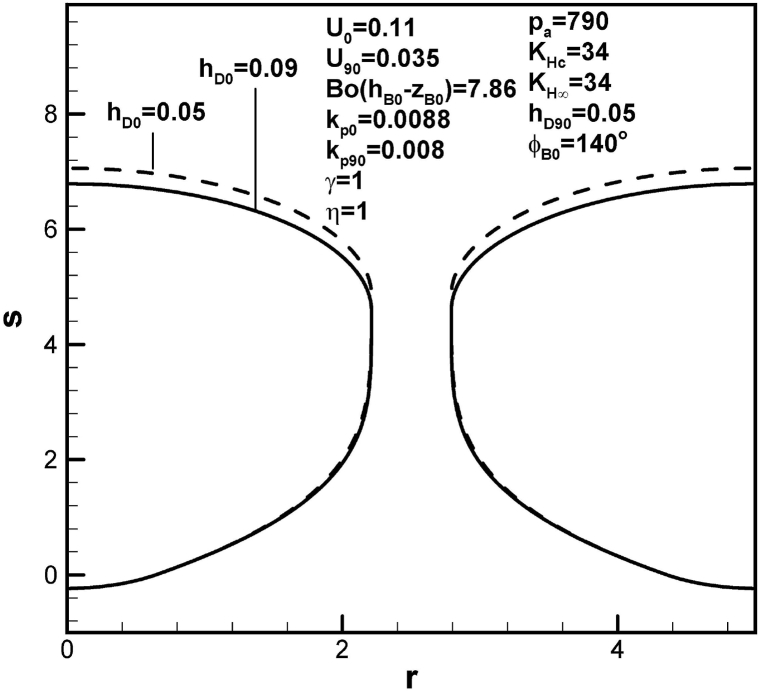
Predicted lotus-type pore shapes affected by dimensionless mass transfer coefficients at time corresponding to initial contact angle.

The effects of dimensionless mass transfer coefficient at initial contact angle on the pore shape are shown in Fig. 8. Dimensionless length of lotus-type pores decreases as dimensionless mass transfer coefficient at initial time increases from 0.05 to 0.09 for mass transfer coefficient at contact angle of 90° of 0.03. This is attributed to a decrease in solute amount in the boundary layer on the bubble cap at initial time.

**Fig. 9:**
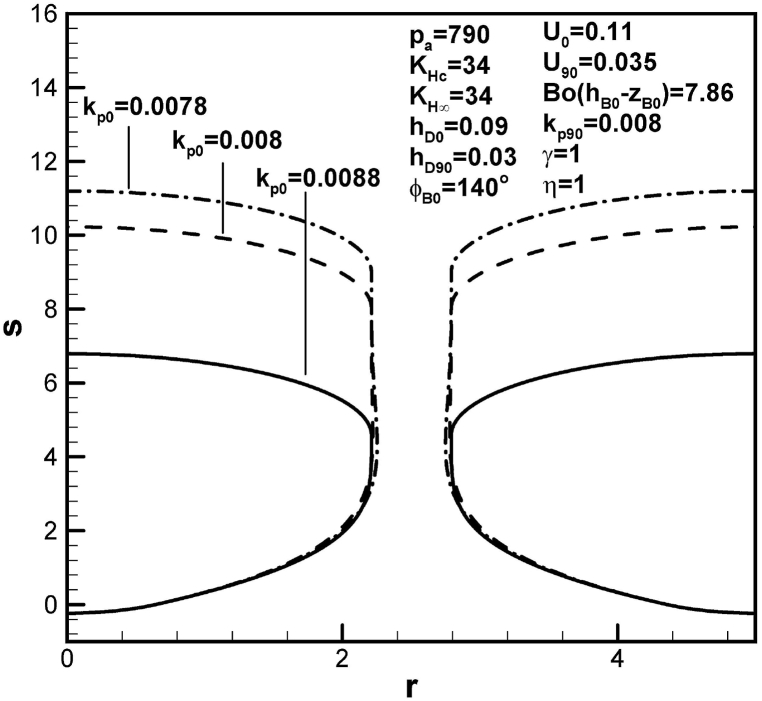
Predicted lotus-type pore shapes affected by partition coefficients at time corresponding to initial contact angle.

The effects of partition coefficient at initial contact angle on the shape of lotus-type pores are shown in Fig. 9. A decrease in partition coefficient at initial contact angle significantly increases the length of the pore, as a result of an increase in solute in the boundary layer on the advancing solid-liquid interface at the initial time.

**Fig. 10:**
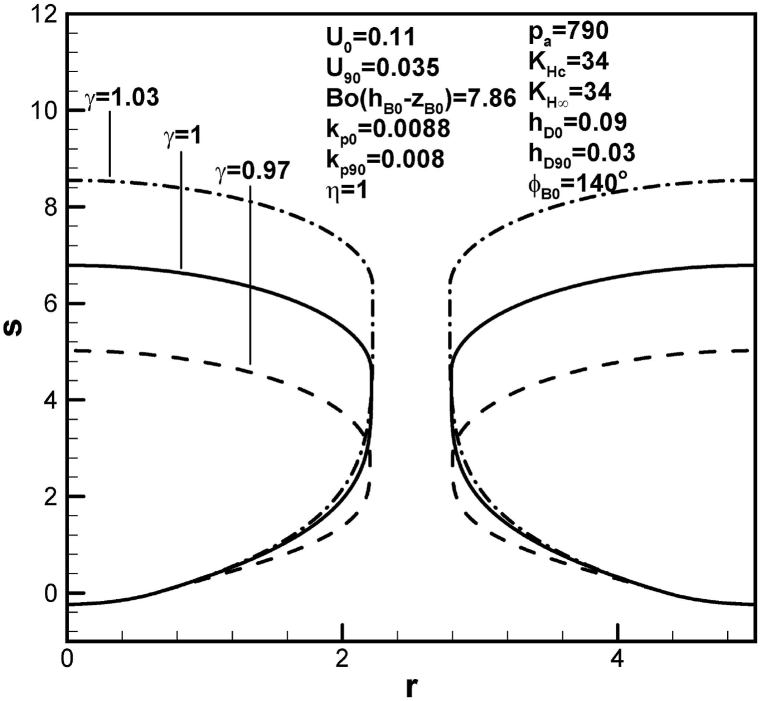
Predicted lotus-type pore shapes affected by mole fraction of solute gas over top surface.

The effects of mole fraction of solute gas at the top free surface on the size of lotus-type pores are shown in Fig. 10. Length of lotus-type pores increases with mole fraction of solute gas on the top free surface. This is attributed to increase in solute amount in the boundary layer on the advancing solid-liquid interface at initial time (see Table 1). It is worthy of mentioning that increase in mole fraction of solute gas on the top free surface decreases solute amount in concentration boundary layer on the cap whereas enhances that on the advancing solid-liquid interface (see Table 1). Mole fraction of solute gas on the top free surface exhibits stronger effects on solute amount in the concentration boundary layer on the solid-liquid interface than that on the bubble cap. The effects of mole fraction of solute gas at the top free surface on solute amount in the concentration boundary layer on the advancing solid-liquid interface at initial time are also stronger than that at contact angle of 90°.

**Fig. 11:**
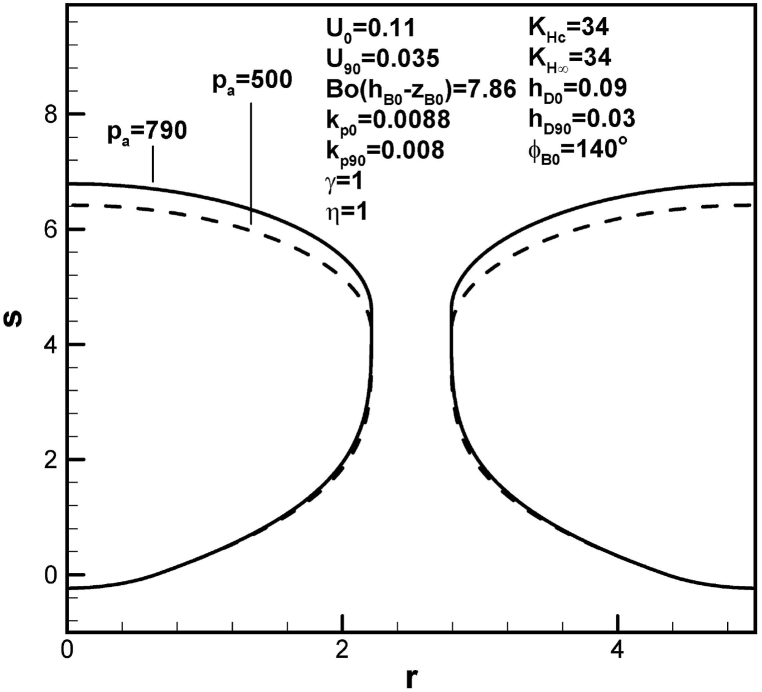
Predicted lotus-type pore shapes affected by dimensionless imposed ambient pressure.

**Fig. 12:**
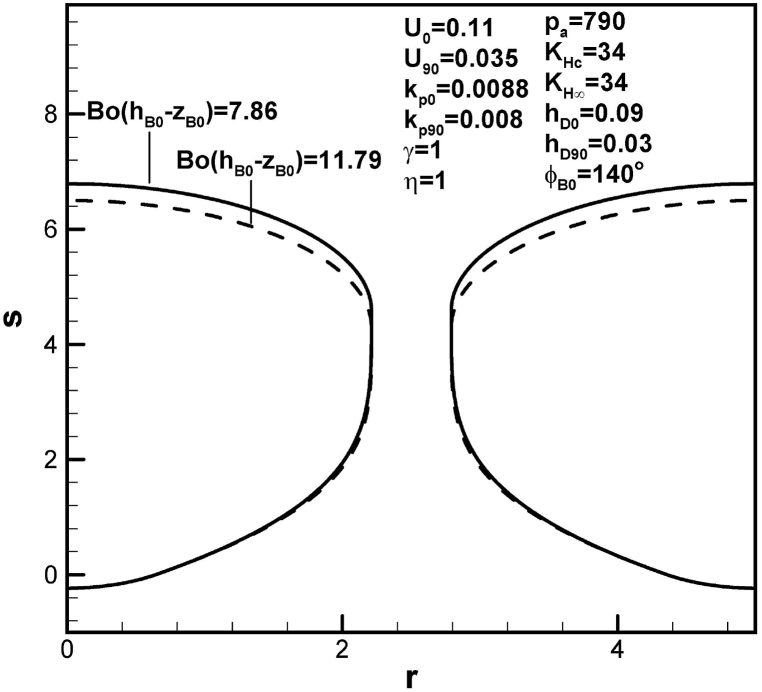
Predicted lotus-type pore shapes affected by dimensionless hydrostatic pressure.

An increase in dimensionless imposed pressure at the top surface increases pore length, as presented in Fig. 11. This is because of an increase in solute amount in the boundary layer on the advancing solid-liquid interface at initial time. An increase in dimensionless hydrostatic pressure decreases dimensionless length of lotus-type pores, as shown in Fig. 12. This is attributed to an increase in solute amount in the boundary layer on the cap at contact angle of 90°.

**Fig. 13:**
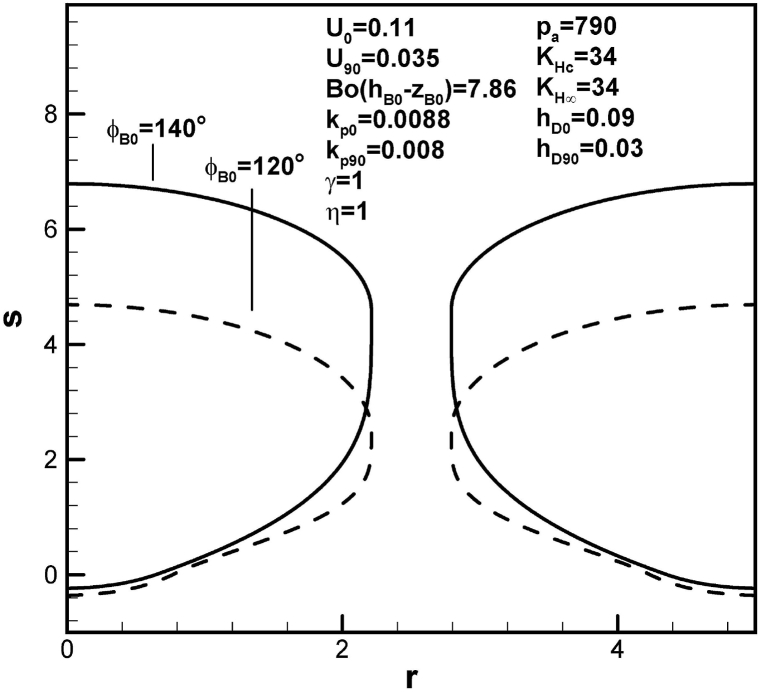
Predicted lotus-type pore shapes affected by initial contact angle.

**Fig. 14:**
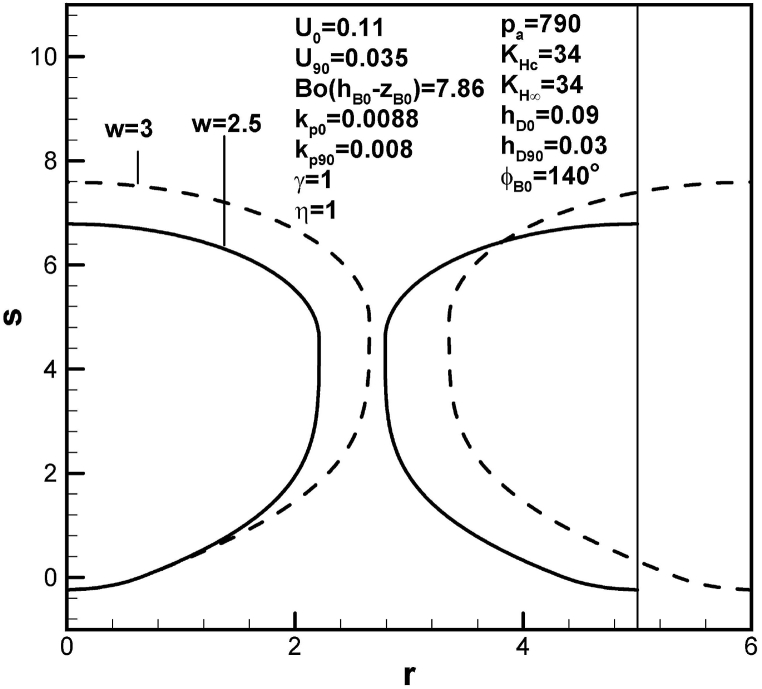
Predicted lotus-type pore shapes affected by dimensionless inter-pore spacing.

**Fig. 15:**
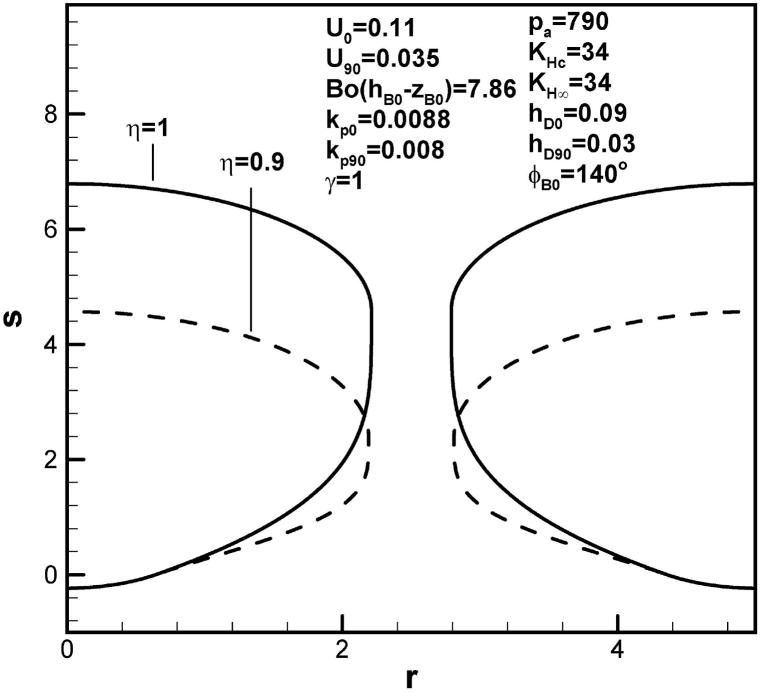
Predicted lotus-type pore shapes affected by solute transport parameter.

Fig. 13, Fig. 14 show that pore length increases with initial contact angle and dimensionless inter-pore spacing. This is a consequence of an increase in solute amount within the boundary layer on the advanced solid-liquid interface at initial time. An increase in solute transport parameter increases radius and length of the pore, as shown in Fig. 15. In view of enhanced solute transport from the boundary layer on the advancing solid-liquid interface into the pore, increase in solute transport parameter decreases solute amount in the boundary layer on the advancing solid-liquid interface at contact angle of 90°.

## 4 Conclusions

The conclusions drawn are as follows:

1. The lotus-type pore shapes characterized by the length and maximum radius affected by working parameters are systematically analyzed from algebraic expressions and Table provided. Prediction and available measurement of diameter and inter-pore spacing during freezing of water dissolved by oxygen gas are in good agreement.
2. Length of lotus-type pores are usually controlled by solute content in the pore and concentration boundary layer on the solidification front. Solute content in the concentration boundary layer on the cap at contact angle of 90° is more important than that at initial time. Solute content in the concentration boundary layer on the initial time is more significant than that at contact angle of 90°.
3. Length and maximum radius exhibit different mechanisms. The former is determined by maintaining total solute amount in the system, composed of the pore and boundary layers on the cap and advancing solid-liquid interface between initial time and time corresponding to contact angle of 90°. The latter is calculated by considering changing rate of solute amount in the pore due to solute transport from boundary layers on the cap and advancing solid-liquid interface at contact angle of 90°.
4. Dimensionless length of lotus-type pores increases with Henry's law constant at the cap. Referring to the Table provided, this is primarily due to decrease in solute amount within the boundary layer on the cap at contact angle of 90°. On the other hand, length of lotus-type pores increases as Henry's law constant at the top surface decreases, resulting from increase in solute amount in the boundary layer on the advancing solid-liquid interface at initial time.
5. Dimensionless length of lotus-type pores increases as solidification rate decreases at initial time. This is because increase in thickness results in increase of solute amount in the boundary layer on the advancing solid-liquid interface at initial time. Similar results can be found for partition coefficients at initial time.
6. Dimensionless length of lotus-type pores increases as dimensionless mass transfer coefficients at initial time decreases. This is because increase in thickness increases solute amount in the concentration boundary layer on the cap at initial time.
7. Dimensionless length of lotus-type pores increases with initial contact angle, dimensionless imposed pressure and mole fraction of solute gas on the top free surface, and inter-pore spacing. This is primarily attributed to enhanced solute amount in boundary layer on the advancing liquid-solid interface at initial time.
8. Dimensionless length of lotus-type pores increases as dimensionless hydrostatic pressure decreases, as a result of decrease in solute amount in the boundary layer on the cap at contact angle of 90°.
9. Dimensionless pore length increases with solute transport parameter, because of decrease in solute within the boundary layer on the advancing solid-liquid interface at contact angle of 90°.

## Author contribution statement

P. S. Wei: Conceived and designed the experiments; Analyzed and interpreted the data; Wrote the paper.

B. Y. Lee: Performed the experiments; Analyzed and interpreted the data; Contributed reagents, materials, analysis tools or data.

## Funding statement

This work is financially supported by NSTC 112-2221-E−110-001, ROC.

## Data availability statement

No data was used for the research described in the article.

## Declaration of competing interest

The authors declare that they have no known competing financial interests or personal relationships that could have appeared to influence the work reported in this paper.

## Nomenclature

*a,b, …,f*: coefficients
*A,B, …,D*: coefficients
*Bo*: Bond number, $Bo\equiv \frac{{\tilde{\rho }}_{l}g{\tilde{R}}_{0}^{2}}{\sigma }$
C: solute concentration, $C=\tilde{C}{\tilde{R}}_{u}{\tilde{T}}_{m}/{\tilde{\rho }}_{l}g{\tilde{R}}_{0}$
*D*: solute diffusivity
$F_{1},F_{2}\text{,}$ …, $F_{5}$: coefficients
*g*: gravitational acceleration
*H*: length scale of system
$h_{B}$: liquid depth
$h_{D}$: mass transfer coefficient, $h_{D}=\frac{{\tilde{h}}_{D}{\tilde{R}}_{0}}{{\tilde{D}}_{l}}$
$K_{H}$: Henry's law constant, $K_{H}=\frac{{\tilde{K}}_{H}}{{\tilde{R}}_{u}{\tilde{T}}_{m}}$
$k_{p}$: partition coefficient
l: length
*Ma*: Marangoni number
*p*: pressure, $p\equiv \tilde{p}{\tilde{R}}_{0}/\sigma$
$p_{a}$: imposed ambient pressure
*Pr*: Prandtl number
*r*: coordinate, $r\equiv \tilde{r}/{\tilde{R}}_{0}$
*R*: apex radius, $R\equiv \frac{\tilde{R}}{{\tilde{R}}_{0}}$
$R_{u}$: universal gas constant
*s*: solidification front location
*Sc*: Schmidt number
*T*: temperature
*U*: solidification rate
*v*: velocity component
*V*: volume, $V=\tilde{V}/{\tilde{R}}_{0}^{3}$
*w*: inter-pore spacing
*z*: coordinate, $z\equiv \tilde{z}/{\tilde{R}}_{0}$

**Greek letter**
α: thermal diffusivity
β: expansion coefficient
δ: boundary layer thickness
σ: surface tension
μ: dynamic viscosity
γ: mole fraction
η: solute transport parameter
ρ: density
ϕ: inclination or contact angle

**Subscripts**
*B*: base
*c*: cap
*C*: concentration
*cr*: critical
*FC*: force convection
*g*: gas
l: liquid
*m*: melting temperature
*NC*: free or natural convection
*R*: apex radius
*s*: solid
*T*: thermal
*0*: initial state
*90*: 90°
∞: far from solidification front

**Superscripts**
∼: dimensional quantity
